# MicroRNA-30a Modulates Type I Interferon Responses to Facilitate Coxsackievirus B3 Replication *Via* Targeting Tripartite Motif Protein 25

**DOI:** 10.3389/fimmu.2020.603437

**Published:** 2021-01-14

**Authors:** Jia Li, Yewei Xie, Liwei Li, Xiaobing Li, Li Shen, Jin Gong, Rufang Zhang

**Affiliations:** ^1^Department of Cardiothoracic Surgery, Shanghai Children’s Hospital, Shanghai Jiaotong University, Shanghai, China; ^2^Shanghai Veterinary Research Institute, Chinese Academy of Agricultural Sciences, Shanghai, China; ^3^Jiangsu Co-Innovation Center for the Prevention and Control of Important Animal Infectious Disease and Zoonose, Yangzhou University, Yangzhou, China

**Keywords:** miR-30a, CVB3, viral replication, TRIM25, type I IFN responses

## Abstract

Viral myocarditis is caused by a viral infection and characterized by the inflammation of the myocardium. Coxsackievirus B3 (CVB3) infection is one of the most common among the infections caused by this virus. The host’s early innate immune response to CVB3 infection particularly depends on the functions of type I interferons (IFNs). In this study, we report that a host microRNA, miR-30a, was upregulated by CVB3 to facilitate its replication. We demonstrated that miR-30a was a potent negative regulator of IFN-I signaling by targeting tripartite motif protein 25 (TRIM25). In addition, we found that TRIM25 overexpression significantly suppressed CVB3 replication, whereas *TRIM25* knockdown increased viral titer and VP1 protein expression. MiR-30a inhibits the expression of TRIM25 and TRIM25-mediated retinoic acid-inducible gene (RIG)-I ubiquitination to suppress IFN-β activation and production, thereby resulting in the enhancement of CVB3 replication. These results indicate the proviral role of miR-30a in modulating CVB3 infection for the first time. This not only provides a new strategy followed by CVB3 in order to modulate IFN-I–mediated antiviral immune responses by engaging host miR-30a but also improves our understanding of its pathogenesis.

## Introduction

Viral myocarditis (VMC) is an inflammatory disease, which appears to be a major cause of sudden cardiac death in children and young adults (1). Coxsackievirus B3 (CVB3) has been considered as the commonest pathogen for VMC (2). Acute VMC is the consequence of myocyte damage induced by early direct virus *via* apoptosis and necrosis, followed by host inflammatory and immune responses (3). Although excessive activation of immune response triggered by virus infection may be a significant cause of tissue injuries, the virus itself is vital to VMC progression *via* direct attack on cardiomyocytes (4, 5). To date, no effective therapies or vaccines for VMC are available currently, although type I interferon (IFN) may show certain efficacy (2, 6). Therefore, studying of the fundamental mechanisms involved in CVB3 infection may provide new clues for researching and developing new therapeutic options against CVB3-induced diseases.

MicroRNAs (miRNAs) are noncoding RNAs that function as gene regulators by repressing translation or degrading target mRNAs (7). The seed region (2–8 nucleotides at the 5′ end) of an miRNA is identified as the key to exerting its silencing function, most commonly by targeting the 5′ or 3′ untranslated region (5′- or 3′-UTR) (8, 9). There is a growing body of evidence that host miRNA-mediated RNA interference is critical in numerous viral infections as negative or positive factors of antiviral immune response (10–12). For example, hepatitis C virus–induced miR-93-5p up-regulation inhibits IFN signaling pathway by targeting interferon alpha receptor 1 (13). MiR-194 facilitated influenza A virus replication by negatively regulating type I IFN production (14). MiR-3570 negatively regulates the retinoic acid-inducible gene (RIG)-I–dependent innate immune response to rhabdovirus by targeting mitochondrial antiviral signaling protein (15). Previous studies suggested that miR-30c regulated macrophage-mediated inflammation and pro-atherosclerosis pathways (16), and miR-30b was identified to be a negative regulator of immune responses by binding Notch1 (17). However, the ability of miR-30 family to modulate CVB3 infection has not been reported.

As an E3 ubiquitin ligase, the tripartite motif protein 25 (TRIM25) is involved in regulating the antiviral immune response (18). Pathogen-associated molecular patterns are immediately recognized by host pattern recognition receptors (PRRs) after virus infection (19). RIG-I is the main cytoplasmic PRR of RNA viruses (20). Then, the caspase recruitment domains (CARDs) of RIG-I are modified by ubiquitin. TRIM25 mediates this progress, which is essential for the activation of type I and III IFNs, mediating viral clearance, and inhibiting viral replication and spread (21, 22). TRIM25 inhibits various viruses, e.g., herpesvirus and influenza virus (23, 24).

We show that host miR-30a could be upregulated upon CVB3 infection. Overexpression of miR-30a exhibits an important role in facilitating CVB3 replication through targeting TRIM25. Mir-30a suppresses TRIM25 expression and TRIM25-mediated RIG-I ubiquitination to inhibit IFN-β activation and production. Together, these findings suggest a novel mechanism through which CVB3 infection suppresses host antiviral immunity by utilizing cellular miRNA and provide insight into the regulatory mechanism between CVB3 and the host.

## Materials and Methods

### Cells and Viruses

HeLa cells and 293T cells were cultured in Dulbecco’s modified Eagle’s medium (DMEM) supplemented with 10% fetal bovine serum (FBS; Gibco, Thermo Fisher Scientific, Waltham, MA). Cells were maintained at 37°C with 5% CO_2_. Primary cardiomyocytes were isolated from one-day-old BALB/c mice (Slac Animal Inc., Shanghai, China) and cultured as described previously (25). The CVB3 (Nancy strain) was stored in our laboratory. CVB3 was maintained by passage through HeLa cells, and cultured in DMEM containing 10% FBS. Virus titer was measured by a 50% tissue culture infectious dose (TCID_50_) assay in HeLa cells.

### miRNA Mimics

miR-30 family mimics (30a, 30b, 30c, 30d, and 30e) were synthesized by GenePharma (Shanghai, China). The sense sequences are listed in **Table 1**. The negative-control (NC) mimic sequence was 5′-uucuccgaacgugucacgutt-3′. The sequences of NC inhibitor (NC-inhi) and miR-30a inhibitor (30a-inhi) are also listed in **Table 1**.

**Table 1:** Sequences of microRNA (miRNA) mimics and inhibitors used in this study.

Name	Sequence (5′−3′)
miR-30a (30a)	UGUAAACAUCCUCGACUGGAAG
miR-30b (30b)	UGUAAACAUCCUACACUCAGCU
miR-30c (30c)	UGUAAACAUCCUACACUCUCAGC
miR-30d (30d)	UGUAAACAUCCCCGACUGGAAG
miR-30e (30e)	UGUAAACAUCCUUGACUGGAAG
miR-30a inhibitor (30a-inhi)	CUUCCAGUCGAGGAUGUUUACA
NC	UUCUCCGAACGUGUCACGUTT
NC inhibitor (NC-inhi)	CAGUACUUUUGUGUAGUACAA

### Quantitative Real-Time PCR

RNAs were extracted using the miRNeasy MiniKit or RNeasy MiniKit (Qiagen, Hilden, Germany) according to the manufacturer’s instructions. For detection of endogenous miRNAs, an miRcute miRNA First-Strand cDNA Synthesis (TIANGEN BIOTECH, Beijing, China) was used for polyadenylation and reverse transcription. A commercial miRcute miRNA qPCR detection kit was used for measuring miRNA abundance. The U6 small nuclear RNA (TIANGEN BIOTECH) was used for normalization. To detect the relative mRNA levels, a PrimeScript RT Master Mix kit (Takara, Dalian, China) was used for reverse transcription. SYBR Premix Ex Taq (Takara) was used to quantify levels of *IFN-α*, *IFN-β*, interferon-stimulated gene 15 (*ISG15*), and *MX1* mRNA. The relative expression levels of these genes were analyzed using the ΔΔCt method (26), and glyceraldehyde-3-phosphate dehydrogenase (*GAPDH*) mRNA was used as a control. Primers are listed in **Table 2**. All PCR experiments were performed in triplicate.

**Table 2:** Sequence of oligonucleotide primers used in this study.

Primer	Sequence (5′−3′)
GAPDH-F	TGGAAATCCCATCACCATCT
GAPDH-R	GGCAGAGATGATGACCCTTT
IFN-α-F	CTCCATTCTGGCTGTGAGGA
IFN-α-R	TGAACCAGTTTTCATTCCTT
IFN-β-F	ACCATCTGAAGACAGTCCTG
IFN-β-R	TCTGACTATGGTCCAGGCAC
ISG15-F	GAAGGCGCAGATCACCCAGA
ISG15-R	GAGGTTCGTCGCATTTGTCC
MX1-F	GGAAGGAATGGGAATCAGTC
MX1-R	TGCCTCTGGATGTACTTCTT

### Dual Luciferase Assays

The pGL3 luciferase reporter plasmids containing the 3′UTR sequence of TRIM25 were cloned downstream of the luciferase open reading frame to test the binding regions in TRIM25. To verify the target gene of miR-30a, 293T cells were co-transfected with 100 ng/well of pRL-TK and 500 ng/well of the indicated reporter plasmid along with the indicated amount of miR-30a or NC mimics. Cells were lysed 24 h post-transfection (hpt) to determine the luciferase activities using a dual-luciferase reporter assay kit (Promega). Data are presented as the relative luciferase activities. To generate the miR-30a target-mutated reporter plasmid (pGL3-TRIM25-mut), mutations at positions corresponding to the miR-30a seed region were introduced. DNA sequencing confirmed mutant plasmid (pGL3-TRIM25-mut) and the primer sequences are available upon request. Luciferase activity in 293T cells were determined. Human IFN-β promoter, NF-κB promoter, and primary miR-30a promoter [a fragment ∼3 kb upstream from the primary miR-30a (pri-miR-30a) coding sequences] plasmids were generated by cloning sequences into the upstream of firefly luciferase gene in the pGL3-Basic vector. To test the promoter activities, HeLa or 293T cells seeded in 24-well plates were co-transfected with the indicated expression plasmids or miRNA mimics along with 100 ng/well of the indicated luciferase reporter plasmid and 20 ng/well of pRL-TK. Twenty-four hpt, luciferase activities were measured and the results are expressed as means ± standard deviation (SD) from three independent experiments.

### Construction of Plasmids

Full-length TRIM25- and RIG-I-encoding sequences were cloned into pCAGGS vector to generate pCAGGS-TRIM25-Myc, pCAGGS-RIG-I-HA, pCAGGS-RIG-I-Flag, or pCAGGS-2CARD-Flag. All plasmids were constructed by homologous recombination using the NEBuilder^®^ HiFi DNA assembly master mix (New England Biolabs; Ipswich, MA). Sequences of all primers will be made available upon request.

### Transfection and Virus Challenge

MiRNA or NC mimics were transfected into HeLa cells at a concentration of 80 nM (except for dosage experiments) using X-tremeGENE siRNA Transfection Reagent (Roche). To investigate the effect of TRIM25 on CVB3 replication, HeLa cells were transfected with 2 μg of pCAGGS-TRIM25-Myc using X-treme GENE HP DNA reagent (Roche). Next, 24 hpt, cells were infected with CVB3 (multiplicity of infection, MOI = 0.1). The supernatants were harvested at 12, 24, and 36 h post-infection (hpi), and the cells were lysed using RIPA lysis buffer (Thermo Fisher Scientific). Viral titers were determined and the amount of viral VP1 was detected by western blotting (WB). WB assays were performed as described previously (27), except using an anti-VP1 antibody (1:1,000) purchased from Leica Biosystems Newcastle Ltd.

### RNA Interference

Small interfering RNAs (siRNAs) against *TRIM25* were synthesized by GenePharma (Shanghai, China). The siRNA molecule sequence was CCU GGA GUA UUA CGU UAA ATT (si-TRIM25). The control siRNA (si-con) sequence was UUC UCC GAA CGU GUC ACG UTT. HeLa cells were transfected with 50 nM si-TRIM25 or si-con using X-tremeGENE siRNA Transfection Reagent (Roche). The cells were lysed 24 hpt, and WB detected the effects of siRNAs using an anti-TRIM25 monoclonal antibody (cat. no. 13773; Cell Signaling Technology; Danvers, MA; 1:1,000). Twenty-four hpt, the cells were infected with CVB3 at an MOI of 0.1. The supernatant and cells were harvested 12 and 24 hpi, and were later analyzed based on virus titers and by WB.

### RISC Immunoprecipitation Assay

An RISC immunoprecipitation assay was performed as previously described (28). 293T cells were transfected with NC or miR-30a mimics using X-tremeGENE siRNA Transfection Reagent (Roche). Cells were harvested 24 hpt. The anti-Ago2 antibody (cat. no. 2897; Cell Signaling Technology) was used for the immunoprecipitation of RISC and the detection of Ago2.

### Statistical Analysis

Statistical analysis was conducted using GraphPad Prism 6 (GraphPad, La Jolla, CA). The statistical significance was analyzed using Student’s *t*-test, with *p* < 0.05 considered statistically significant.

## Results

### miR-30a Is Up-Regulated in Response to CVB3 Infection *In Vitro*

**Figure 1:**
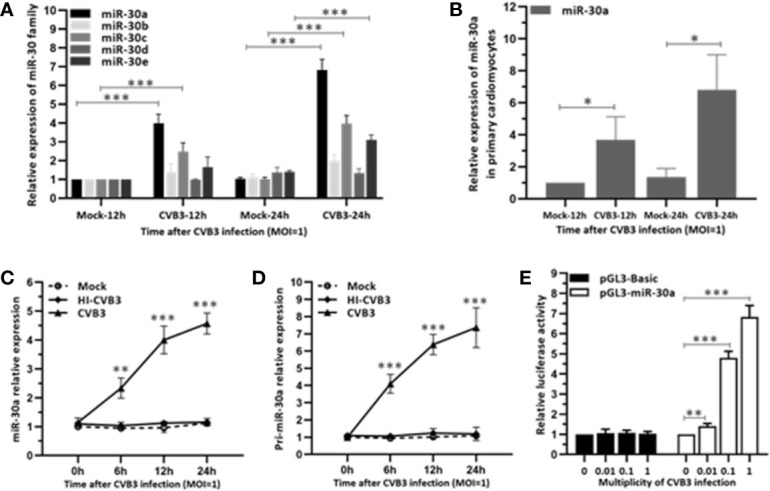
miR-30a is up-regulated in response to CVB3 infection *in vitro*. **(A)** qRT-PCR analysis of miR-30 family was performed in HeLa cells inoculated with CVB3 at an MOI of 1 for the indicated times. **(B)** Primary cardiomyocytes of mice were mock infected or infected by CVB3 (MOI = 1) for 12 and 24 h. The relative level of miR-30a was analyzed by qRT-PCR analysis. **(C)** qRT-PCR analysis of miR-30a was performed in HeLa cells inoculated with HI-CVB3 (MOI = 1) at 0, 6, 12, and 24 hpi. **(D)** qRT-PCR analysis of pri-miR-30 was performed in HeLa cells inoculated with CVB3 (MOI = 1) at 24 hpi. **(E)** After co-transfection of 100 ng pGL3-basic or pGL3-miR-30a promoter along with pRL-TK (20 ng) into HeLa cells for 12 h, cells were infected with CVB3 at an MOI of 0.01, 0.1, or 1. At 24 hpi, luciferase activities were analyzed. Data represent the mean ± SD of three independent experiments. Statistical significance was analyzed using *t* tests. *P < 0.05; **P < 0.01; ***P < 0.001.

To investigate whether miR-30 family can be regulated by CVB3, we performed a time-course assay to analyze the expression of the miR-30 family members in HeLa cells infected with CVB3 (MOI = 1) or mock infected. The miR-30 family contains five members (miR-30a–e). Results indicated that miR-30a was significantly increased at 12 hpi and peaked at 24 hpi (∼6.6-fold induction) and the expression of other miR-30 family members showed slight alteration (**Figure 1A**). To confirm that the target cells of CVB3 function equivalently, primary cardiomyocytes were cultured from newborn mice and infected with CVB3 (MOI = 1). Results showed that CVB3 infection enhanced the relative expression of miR-30a at 12 and 24 hpi, thereby demonstrating that miR-30a expression is upregulated by CVB3 infection, which may be involved in regulating CVB3 infection (**Figure 1B**). Then, Hela cells were infected with CVB3 or heat-inactivated CVB3 (HI-CVB3; 70°C for 60 min) at MOI of 0.1. The expression of miR-30a in the cells inoculated with HI-CVB3 was not altered (**Figure 1C**), suggesting that the increased miR-30a depends on CVB3 replication.

To verify the induction of miR-30a by CVB3 infection, the expression of its primary transcript pri-miR-30a was analyzed. We demonstrated that pri-miR-30a was significantly upregulated and reached a significant increment to ∼7.2-fold at 24 hpi (**Figure 1D**). To corroborate the findings further, pri-miR-30a promoter was transfected into HeLa cells, followed by CVB3 infection. CVB3 infection enhanced the activity of the miR-30a promoter with increasing MOIs, and it increased ∼4.7- and ∼6.8-fold at MOIs of 0.1 and 1, respectively (**Figure 1E**).

### miR-30a Significantly Facilitates CVB3 Replication

**Figure 2:**
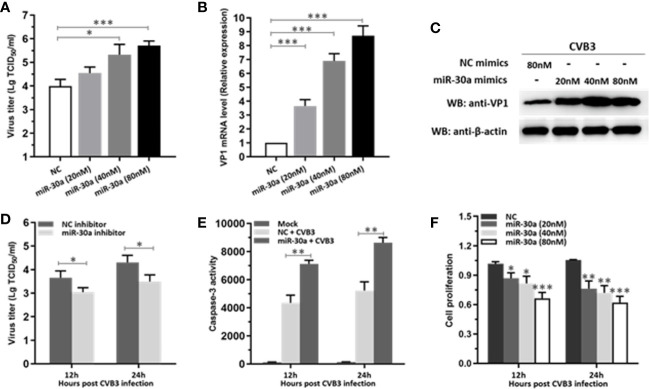
miR-30a significantly facilitates CVB3 replication. **(A)** HeLa cells were transfected with miR-30a or NC mimics at the indicated doses (20–80 nM), followed by CVB3 infection (MOI = 0.1). The supernatants were collected at the indicated times for viral titer determination. **(B)** qRT-PCR analysis of VP1 mRNA levels in HeLa cells transfected with miR-30a or NC mimics at the indicated doses (20–80 nM), followed by CVB3 infection (MOI = 0.1). **(C)** The experiments were performed as described for panel **(B)**. Cells were collected at 24 hpi for WB analysis of VP1 expression. β-actin expression was analyzed as a loading control. **(D)** HeLa cells were transfected with miR-30a inhibitor or NC inhibitor (80 nM), followed by CVB3 infection (MOI = 0.1). Supernatants were collected at 12 and 24 hpi for viral titer determination. **(E)** HeLa cells were with miR-30a or NC mimics, followed by CVB3 infection (MOI = 0.1). The caspase-3 activity was detected at 12 and 24 hpi. **(F)** Cell viability assay was performed at 12 and 24 hpi with the indicated dose of miR-30a or NC mimics. The viability was tested by MTT assay. Data represent the mean ± SD of three independent experiments. Statistical significance was analyzed using *t* tests. *P < 0.05; **P < 0.01; ***P < 0.001.

To verify whether miR-30a can affect CVB3 replication, the expression of CVB3 capsid protein VP1 and viral titers in HeLa cells at 24 hpi were detected. Both CVB3 growth and the amount of VP1 mRNA level were upregulated as a function of the dose of miR-30a mimics (**Figures 2A, B**). Consistent with this, transfecting the miR-30a mimics induced the accumulation of VP1 (**Figure 2C**). Results above suggest that viral replication and progeny release in HeLa cells were significantly increased by miR-30a. To illustrate the proviral effect of miR-30a on CVB3 replication, we analyzed viral infection in the presence of the miR-30a inhibitor. MiR-30a inhibitor significantly reduced virus titer to that of NC inhibitor at 12, and 24 hpi, respectively (**Figure 2D**). This indicated that miR-30a promoted CVB3 replication in HeLa cells.

We next sought to investigate the effect of miR-30a on apoptosis post CVB3 infection. As shown in **Figure 2E**, CVB3 infection caused a dramatic increase in the caspase-3 activity in miR-30a-transfected cells than that in NC-transfected cells, suggesting a potential promoting role of miR-30a for CVB3-induced apoptosis in infected cells. Indeed, miR-30a significantly promoted the proliferation in CVB3-infected cells in a dose- and time-dependent manner (**Figure 2F**), indicating that miR-30a also aggravated CVB3‐induced apoptosis. Taken together, these results suggest that miR-30a promoted CVB3 replication and aggravated CVB3‐induced apoptosis in HeLa cells.

### miR-30a Represses Type I IFN Activation and Production

**Figure 3:**
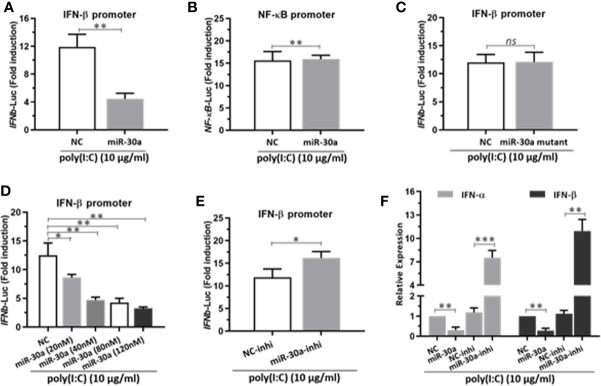
miR-30a represses IFN-α/β activation and production. **(A)** IFN-β or **(B)** NF-κB promoter vector along with pRL-TK were co-transfected with 80 nM of the miR-30a or NC mimics into 293T cells for 24 h, followed by stimulation with poly(I:C) (10 μg/ml) for 12 h. Then, 293T cells were harvested for luciferase assay. **(C–E)** The experiments were performed as described for panel **(A)**, except that miR-30a mutant, the indicated doses (20–120 nM) of miR-30a mimics, or miR-30a inhibitor were used. **(F)** qRT-PCR analysis of type I IFN α/β mRNA expression in HeLa cells transfected with NC mimics, miR-30a mimics, NC inhibitor, or miR-30a inhibitor for 24 h, followed by stimulation with poly(I:C) (10 μg/ml) for 12 h. Data were normalized to GAPDH expression and are the mean ± SD of three independent experiments. Statistical significance was analyzed using *t* tests. *P < 0.05; **P < 0.01; ***P < 0.001.

Next, to investigate the potential mechanisms for miR-30a to enhance CVB3 infection, we tested whether miR-30a can alter NF-κB and IFN-I activation. We performed a series of assays to monitor the activation of NF-κB promoter and IFN-β promoter after stimulating with poly(I:C). Our data indicated that miR-30a significantly repressed the activation of IFN-β under poly(I:C) stimulation (**Figure 3A**), whereas it had no effect on the activation of NF-κB promoter (**Figure 3B**). However, miR-30a mutant with mutated seed sequences (GUAAACA to GAUUUGA) lost its ability to inhibit the activation of IFN-β (**Figure 3C**). Results above suggest that miR-30a regulates type I IFN activation. The inhibition of IFN-β activation by miR-30a occurred in a dose-dependent manner (**Figure 3D**), whereas miR-30a inhibitor enhanced IFN-β activation (**Figure 3E**). To confirm these results, we found that the relative mRNA levels of IFN-α/β expression decreased in miR-30a-transfected cells, whereas miR-30a inhibitors had the opposite effect (**Figure 3F**). These results demonstrated the role of miR-30a as a negative regulator of type I IFN activation and production.

### miR-30a Inhibits the Expression of TRIM25 Through Targeting the 3′UTR of TRIM25

**Figure 4:**
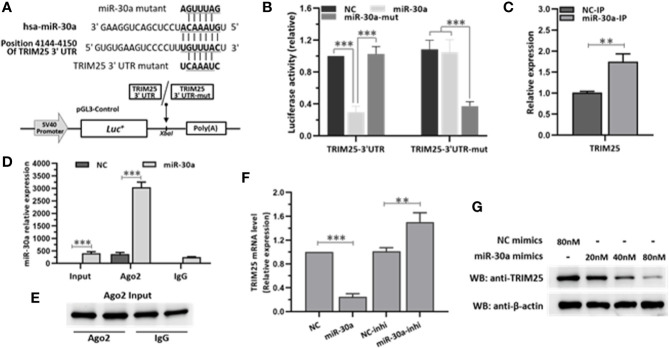
miR-30a inhibits the expression of TRIM25 by targeting the 3′UTR of TRIM25. **(A)** Computational prediction of potential miR-30a target sites in the 3′UTR of TRIM25. The predicted target sites of miR-30a are underlined and were mutated as indicated. The fragments were inserted in *XbaI* site. **(B)** Luciferase activity in lysates of 293T cells co-transfected with TRIM25-3′UTR or TRIM25-3′UTR-mutant luciferase reporter vector and miR-30a mimics or NC mimics. **(C–E)** 293T cells were transfected with miR-30a or NC mimics (80 nM) for 24 h. Cells were analyzed by RISC-IP followed by qRT-PCR to analyze *TRIM25* (normalized to *GADPH*) **(C)**, or miR-30a compared to RNAs extracted from total samples of lysates from 293T cells (normalized to *U6*) **(D)** or WB to analyze Ago2 expression from input **(E)**. **(F)** qRT-PCR analysis of TRIM25 mRNA levels in 293T cells transfected with miR-30a mimics or miR-30a inhibitor (80 nM). Data are presented as mean ± SD. Statistical significance was analyzed using *t*-tests. **P < 0.01; ***P < 0.001. **(G)** WB analysis of TRIM25 expression levels in cells transfected with miR-30a mimics at the indicated doses (20–80 nM). β-actin expression was analyzed as a loading control.

To investigate the major target of miR-30a that is involved in modulating IFN-I responses, we used TargetScan program (http://www.targetscan.org) to predict the target genes. Analysis showed that miR-30a could target TRIM25 through a site in the 3′UTR region (**Figure 4A**). To validate whether TRIM25 is a direct target of miR-30a, the cDNA fragments containing the predicted target site were constructed into a firefly luciferase reporter vector pGL3-Control; meanwhile, a mutant vector was constructed to eliminate the recognition by replacing five seed nucleotides (UGUUUAC to UCAAAUC). In the presence of miR-30a, the luciferase activity of TRIM25 3′UTR was reduced to ∼29% relative to NC. However, the effects produced by miR-30a were eliminated in the vector bearing the mutant target site (**Figure 4B**). These results confirmed that the miR-30a target site is harbored in the 3′UTR of TRIM25. To clarify that miR-30a targets TRIM25 mRNA directly by binding to its 3′UTR region, the cells were transfected with miR-30a for 24 h and then lysed and harvested for the pull-down of miR-30a and TRIM25 mRNA by anti-Ago2 monoclonal antibody. Quantitative real-time PCR (qRT-PCR) data showed that the transfection of miR-30a increased the fraction of TRIM25 mRNA bound by Ago2, and the enrichment efficiency was about 1.8-fold (**Figure 4C**). At the same time, miR-30a associated with Ago2 was significantly enriched by about 7.6-fold in the miR-30a–overexpressed sample compared to NC (**Figure 4D**). As Ago2 had a similar input among different treatments (**Figure 4E**), we concluded that miR-30a physically bound TRIM25 mRNA along with Ago2 in RISC. The results suggest that miR-30a physically binds to the 3′UTR region of TRIM25 mRNA. To further verify TRIM25 as a target of miR-30a, its expression was tested in 293T cells transfected with miR-30a mimics or inhibitors. The mRNA levels of TRIM25 were decreased when miR-30a was overexpressed but were increased when the miR-30a inhibitor was applied (**Figure 4F**). MiR-30a mimics significantly inhibited the level of TRIM25 protein in a dose-dependent manner (**Figure 4G**). Taken together, results above demonstrate that TRIM25 is a target of miR-30a.

### TRIM25 Restricts CVB3 Replication by Upregulating IFN-β Activation and Production

**Figure 5:**
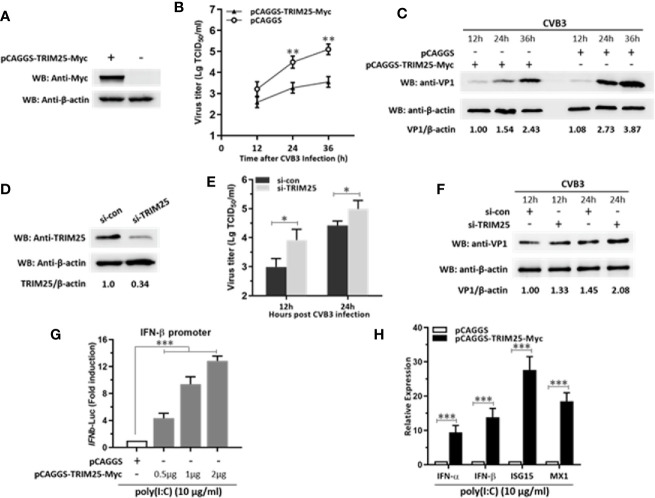
TRIM25 restricts CVB3 replication by upregulating IFN-β activation and production. **(A)** HeLa cells were transiently transfected with pCAGGS-TRIM25-Myc or pCAGGS. 24 hpt, cells were subjected to WB for detecting TRIM25 expression levels. **(B, C)** HeLa cells were transiently transfected with pCAGGS-TRIM25-Myc or pCAGGS. 24 hpt, cells were infected with CVB3 at an MOI of 0.1. Supernatants were collected and subjected to TCID_50_ **(B)** and WB analyzed VP1 levels **(C)**. **(D)** HeLa cells were transfected with control siRNA (si-con) or TRIM25 siRNA (si-TRIM25) and the knockdown efficiency was analyzed by WB at 24 hpt. **(E, F)** Cells were transfected with si-con or si-TRIM25 (50 nM) for 24 h and infected with CVB3 (MOI = 0.1). Cells and supernatants were harvested at the indicated times. **(E)** TCID50 assay and **(F)** WB analysis evaluated CVB3 replication. **(G)** IFN-β promoter vector along with pRL-TK were co-transfected with pCAGGS-TRIM25-Myc or pCAGGS into cells for 24 h, followed by stimulation with poly(I:C) (10 μg/ml) for 12 h. Then, cells were harvested for luciferase assay. **(H)** qRT-PCR analysis of IFN-α, IFN-β, MX1, and ISG15 expression in cells transfected with pCAGGS-TRIM25-Myc or pCAGGS for 24 h and then infected with CVB3 for 24 h at an MOI of 0.1. Data were normalized to GAPDH expression and are the mean ± SD of three independent experiments. Statistical significance was analyzed using *t* tests. *P < 0.05; **P < 0.01; ***P < 0.001.

To investigate the role of TRIM25 on CVB3 replication, HeLa cells were transfected with a plasmid expressing TRIM25 or vector alone and then infected with CVB3 at MOI of 0.1. The efficiency of overexpression of TRIM25 was confirmed by WB (**Figure 5A**). The supernatants were subjected to TCID_50_ assay to determine the role of TRIM25 on viral progeny release. As shown in **Figure 5B**, TRIM25 overexpression significantly reduced the virus particle release. Furthermore, the protein level of VP1 was significantly inhibited by TRIM25 overexpression (**Figure 5C**). To verify the antiviral effect of TRIM25, we designed and screened a specific siRNA targeting the open reading frame of TRIM25, which led to a 70% reduction in the overall levels of the TRIM25 protein (**Figure 5D**). Knockdown of TRIM25 significantly increased CVB3 progeny production (**Figure 5E**) and viral capsid protein VP1 expression (**Figure 5F**), compared to the effect of control siRNA. Collectively, the results confirmed that TRIM25 significantly restricted CVB3 replication *in vitro*. To confirm IFN-β promoting role of TRIM25, HeLa cells were co-transfected with TRIM25 vector and IFN-β promoter-luciferase plasmid. As demonstrated in **Figure 5G**, overexpression of TRIM25 enhanced the activity of IFN-β promoter in a dose-dependent manner after CVB3 infection. Furthermore, TRIM25 significantly increased the IFN-β, ISG15, and MX1 mRNA levels upon CVB3 infection (**Figure 5H**). Thus, our data suggested that TRIM25 suppresses CVB3 replication by upregulating the activation of IFN signaling pathway.

### mir-30a Suppresses TRIM25-Mediated RIG-I Ubiquitination to Suppress IFN-β Activation and Production

**Figure 6:**
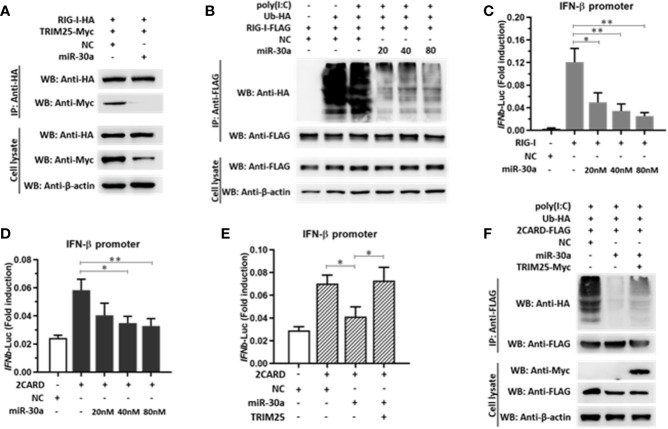
miR-30a suppresses TRIM25-mediated RIG-I activation and IFN production. **(A)** pCAGGS-RIG-I-HA and pCAGGS-TRIM25-Myc were co-transfected with or without miR-30a. The interaction was probed by immunoprecipitation. **(B)** 293T cells were co-transfected with the indicated plasmids and miR-30a or NC mimics for 36 h, and were treated with or without poly(I:C) (10 μg/ml) for 12 h. Anti-Flag immunoprecipitates prepared from the cell extracts were analyzed by WB using the indicated antibodies. **(C, D)** 293T cells seeded in 24-well plates were co-transfected using the IFN-β-Luc firefly luciferase reporter plasmid and the Renilla luciferase control reporter plasmid pRL-TK. For the experiment, pCAGGS-RIG-I-Flag or pCAGGS-2CARD-Flag were co-transfected with or without miR-30a. **(E)** pCAGGS-2CARD-Flag (0.25 μg) and pCAGGS-TRIM25-Myc (0.5 μg) plasmids with or without miR-30a were co-transfected. The luciferase activity in cell lysates was measured. The data are presented as the mean ± SD from three experiments. Statistical significance was analyzed using *t* tests. *P < 0.05; **P < 0.01. **(F)** Ubiquitin-HA (0.5 μg), pCAGGS-2CARD-Flag (0.5 μg), or pCAGGS-TRIM25-Myc (1.0 μg) were co-transfected with or without miR-30a. 24 hpt, cells were treated with poly(I:C) (10 μg/ml) for 12 h, cell lysates were analyzed by immunoprecipitation using the indicated antibodies to detect the ubiquitination of RIG-I-2CARD.

TRIM25-mediated RIG-I ubiquitination is essential for the activation of type I and III IFNs. In the current study, we demonstrated that miR-30a significantly suppressed the expression of TRIM25. Whether miR-30a can suppress TRIM25-mediated RIG-I activation was unknown. We first verified the effect of miR-30a overexpression on the interaction between RIG-I and TRIM25. When RIG-I-HA and TRIM25-Myc were co-transfected with miR-30a mimics, the interaction between TRIM25 and RIG-I was markedly diminished. Meanwhile, when TRIM25-Myc and RIG-I-HA were co-transfected with NC mimics, the interaction between TRIM25 and RIG-I was not affected (**Figure 6A**). To investigate whether TRIM25-mediated RIG-I ubiquitination is regulated by miR-30a, 293T cells were co-transfected with pCAGGS-Flag-RIG-I (0.5 μg per well) and HA-ubiquitin (0.5 μg per well), and the indicated dose of miR-30a mimics. The experiment revealed that TRIM25-mediated RIG-I ubiquitination was potentiated under poly(I:C) stimulation but was substantially suppressed by increasing miR-30a in a dose-dependent manner (**Figure 6B**). To examine regulation of RIG-I activity by miR-30a, a luciferase reporter under the control of the *IFN-β* promoter was used to quantify promoter activation. Consistent with the inhibition of RIG-I ubiquitination by miR-30a, *IFN-β* promoter activation induced by RIG-I or RIG-I CARD domain overexpression was significantly inhibited by miR-30a in a dose-dependent manner (**Figures 6C, D**). However, co-expression of TRIM25 with miR-30a counteracted this inhibitory effect mediated by miR-30a (**Figure 6E**). In addition, suppression of RIG-I ubiquitination by miR-30a was partially rescued by TRIM25 overexpression (**Figure 6F**). These observations indicated that the miR-30a inhibits TRIM25-mediated RIG-I ubiquitination to suppress IFN-β activation and production.

## Discussion

Recently, host miRNAs emerged as key regulators and potential drug targets in CVB3 infection. miR‐221 and miR‐155 have been reported to participate in a CVB3‐induced inflammatory response (29, 30). miR‐34a aggravates CVB3‐induced apoptosis of cardiomyocytes through the SIRT1‐p53 pathway (31). MiR-223/Pknox1 axis protects mice from CVB3-induced VMC by modulating macrophage polarization (32). miRNA-21 shows protective role against CVB3 infection by targeting the MAP2K3/P38 MAPK signaling pathway (33). In this study, we demonstrated for the first time that miR‐30a participates in the pathogenesis during CVB3 infection *in vitro*.

Previous studies report that miR-30c facilitated porcine reproductive and respiratory syndrome virus infection by targeting JAK1 and IFN-alpha/beta receptor beta chain (34, 35). However, influenza A virus restrained host type I IFN-mediated antiviral immune responses by decreasing the expression of miR-30 family members *via* targeting SOCS1 and SOCS3 expression (36).

In this study, we showed that miR-30a functions by facilitating CVB3 replication, and that it could act as a host-derived promoter of the virus (**Figure 2**). Furthermore, miR-30a facilitates CVB3 infection by repressing IFN-I signaling and by targeting TRIM25 (**Figures 3** and **4**). As an E3 ubiquitin ligase, TRIM25 can regulate the antiviral immune response (22). TRIM25-mediated ubiquitination of the cytosolic PRR RIG-I is an essential step for the initiation of an intracellular antiviral response (37). Data presented (**Figure 5**) revealed that upon TRIM25 overexpression, CVB3 replication was significantly suppressed by upregulating type I IFNs and interferon-stimulated genes (ISGs) production. However, in the course of natural infection, CVB3 can complete the replication cycle and efficiently spread. Hence, CVB3 has evolved several general strategies to suppress host innate immunity therefore does not cause robust IFN responses and ISG expressions. In the current study, miR-30a was significantly up-regulated in response to CVB3 infection in HeLa cells and cardiomyocytes, thereby suggesting that miR-30a may be involved in regulating of CVB3 infection and the progression of VMC or other CVB3-induced diseases (**Figure 1**). Mir-30a suppressed TRIM25 expression and TRIM25-mediated RIG-I ubiquitination to suppress IFN-β activation and production (**Figure 6**). This might be the mechanism adopted by CVB3 to antagonize the antiviral response of host.

In the present study, we confirmed that miR-30a modulates type I IFN responses to facilitate CVB3 replication *via* targeting TRIM25. Furthermore, CVB3 can antagonize the antiviral activity of TRIM25 by increasing miR-30a level *in vitro*. These results improve the understanding of the effect of miR-30a on CVB3 replication and the mechanism by which CVB3 evades the TRIM25-mediated innate immune response. Hence, the current study not only offers a novel mechanism through which miR-30a inhibits host innate immunity but also provides improved understanding of the mutual regulatory mechanism between CVB3 and the host innate immune responses.

## Data Availability Statement

The raw data supporting the conclusions of this article will be made available by the authors, without undue reservation.

## Author Contributions

This study was conceived and designed by RZ. All authors participated in the experiments. JL wrote the main manuscript text and prepared the figures. LL and YX performed the experiments. XL, LS, and JG prepared the manuscript. All authors contributed to the article and approved the submitted version.

## Funding

This work was supported by the Shanghai Science and Technology Innovation Action Plan (17411969000) and the Foundation of Key Laboratory of Veterinary Biotechnology (No. shklab202004), Shanghai, P.R. China.

## Conflict of Interest

The authors declare that the research was conducted in the absence of any commercial or financial relationships that could be construed as a potential conflict of interest.
